# Hemisphere-asymmetric tropical cyclones response to anthropogenic aerosol forcing

**DOI:** 10.1038/s41467-021-27030-z

**Published:** 2021-11-22

**Authors:** Jian Cao, Haikun Zhao, Bin Wang, Liguang Wu

**Affiliations:** 1 Key Laboratory of Meteorological Disaster, Ministry of Education/Joint International Research Laboratory of Climate and Environment Change/Collaborative Innovation Center on Forecast and Evaluation of Meteorological Disasters, Nanjing University of Information Science and Technology, Nanjing, China; 2 Earth System Modeling Center, Nanjing University of Information Science and Technology, Nanjing, China; 3 Department of Atmospheric Sciences, University of Hawaii at Mānoa, Honolulu, HI USA; 4 Department of Atmospheric and Oceanic Sciences and Institute of Atmospheric Sciences, Fudan University, Shanghai, China

**Keywords:** Atmospheric dynamics, Climate change

## Abstract

How anthropogenic forcing could change tropical cyclones (TCs) is a keen societal concern owing to its significant socio-economic impacts. However, a global picture of the anthropogenic aerosol effect on TCs has not yet emerged. Here we show that anthropogenic aerosol emission can reduce northern hemisphere (NH) TCs but increase southern hemisphere (SH) TCs primarily through altering vertical wind shear and mid-tropospheric upward motion in the TC formation zones. These circulation changes are driven by anthropogenic aerosol-induced NH-cooler-than-SH and NH-increased versus SH-decreased meridional (equator to mid-latitudes) temperature gradients. The cooler NH produces a low-level southward cross-equatorial transport of moist static energy, weakening the NH ascent in the TC formation zones; meanwhile, the increased meridional temperature gradients strengthen vertical wind shear, reducing NH TC genesis. The opposite is true for the SH. The results may help to constrain the models’ uncertainty in the future TC projection. Reduction of anthropogenic aerosol emission may increase the NH TCs threat.

## Abstract (web summary)

The impact of anthropogenic emissions on climate dynamics is important in our changing climate. Here, the authors show that anthropogenic aerosol emissions can reduce northern hemisphere tropical cyclones but will increase in the southern hemisphere primarily through altering vertical wind shear and upward motion in the tropical cyclone formation zones.

## Introduction

Tropical cyclone (TC) intensity, precipitation, and the accompanying storm surges and floods are projected to increase with anthropogenic forcing-induced global warming^1–3^. However, whether anthropogenic forcings have yet affected the statistics of TCs and how anthropogenic emissions will change future global TC frequency remain inconclusive^4–8^. One of the main obstacles is the lack of understanding of how individual anthropogenic forcing alters TC activity^8,9^.

Anthropogenic aerosol, one of the leading anthropogenic forcings^10^, is emitted mainly from the NH continents and alters regional circulation significantly^11,12^. Previous studies have examined anthropogenic aerosol’s impacts on regional-scale TC frequency over the individual basins of the NH, including North Atlantic^13,14^, Northwestern Pacific^15^, and North Indian Ocean^16^. The regional TCs respond sensitively to the NH-concentrated, spatially, and temporally variable anthropogenic aerosol emission^17^. However, it is still unknown whether the anthropogenic aerosol alters the global TC frequency, although aerosols can change the global climate by mediating sea surface temperature changes^18,19^. Revealing its global impact on TCs can provide a global view to unify its local and remote impacts in individual ocean basins. Thus far, how and why anthropogenic aerosol changes global-scale TCs remains a gap of knowledge.

Some studies have noticed that a global-scale change in TC activity can be caused by the natural aerosols released from desert^20^ or volcano eruptions^21,22^ and by solar radiation change induced by Earth-orbital variation^23,24^ shifting intertropical convergence zone (ITCZ) or Hadley circulation. But, how the detailed physical processes responsible for the TC formation-related environment changes were less explored. The anthropogenic aerosol differs from the natural solar-volcanic forcing in terms of its spatial source distribution and the ways affecting sea surface temperature and atmospheric general circulation^17–19,22,25^. An improved understanding of the mechanisms by which anthropogenic aerosol affects global-scale TCs is expected to build up the connection between NH human activity to the global TC disasters and to project future TC changes more reliably.

In this work, we examine how the anthropogenic aerosol forcing redistributes the global TC frequency using the historical aerosol-forced experiment^26^. We find that the anthropogenic aerosol can induce the hemispheric temperature differences: NH is cooler than SH, and the NH increases the north-south temperature gradient while SH decreases it. The aerosol-forced temperature pattern causes the descent motion and increased vertical wind shear over the NH but ascent motion and decreased vertical wind shear over the SH. As a result, the TC frequency is suppressed over the NH and increased over the SH, forming a hemisphere-asymmetric TC frequency pattern.

## Results

### NH-decreased and SH-increased TCs forced by the anthropogenic aerosol

We used the historical aerosol-forced (hist-aer) simulations (Methods) from 13 coupled model intercomparison project phase 6 (CMIP6) models (Supplementary Table 1) to investigate the impact of anthropogenic aerosol forcing on global TCs. The influence of anthropogenic aerosol is represented by the difference between the present (1985-2014) and the pre-industrial period (1850–1879) climatology. We use two TC genesis potential indices (GPIs), Emanuel-Nolan’s GPI^27^ (ENGPI hereafter) and dynamic GPI (DGPI)^28^, as the proxies of TC formation to investigate what factors determine the global-scale TCs’ generation (Methods). Both GPIs represent the observed modern TC climatology very well (Supplementary Fig. 1).

**Fig. 1:**
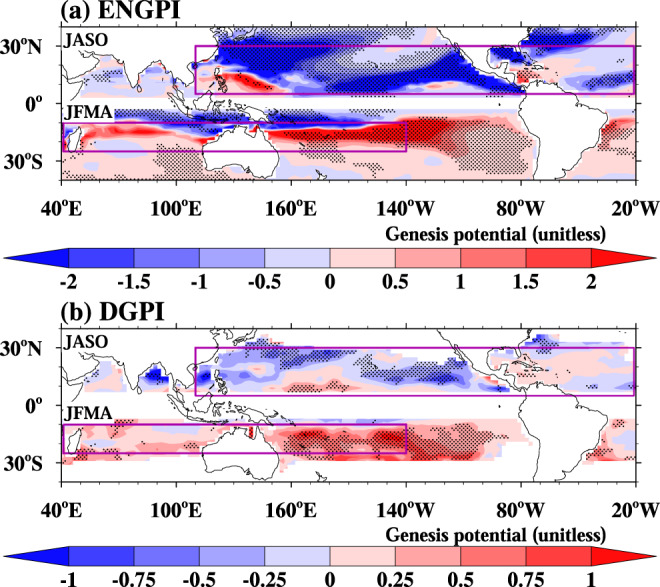
Tropical cyclone (TC) genesis frequency change inferred by two empirical TC genesis potential indices (GPIs). **a** Emanuel-Nolan’s genesis potential index (ENGPI). **b** dynamic GPI (DGPI). Shown are the July-October (JASO) change over the Northern Hemisphere and January-April (JFMA) change over the Southern Hemisphere between the modern period (1985–2014) and pre-Industrial period (1850–1879) from 13 CMIP6 models, respectively. Stippled regions indicate the sign changes are significant at a 95% bootstrap confidence. The purple boxes present the TC formation zones over the NH and SH.

**Fig. 2:**
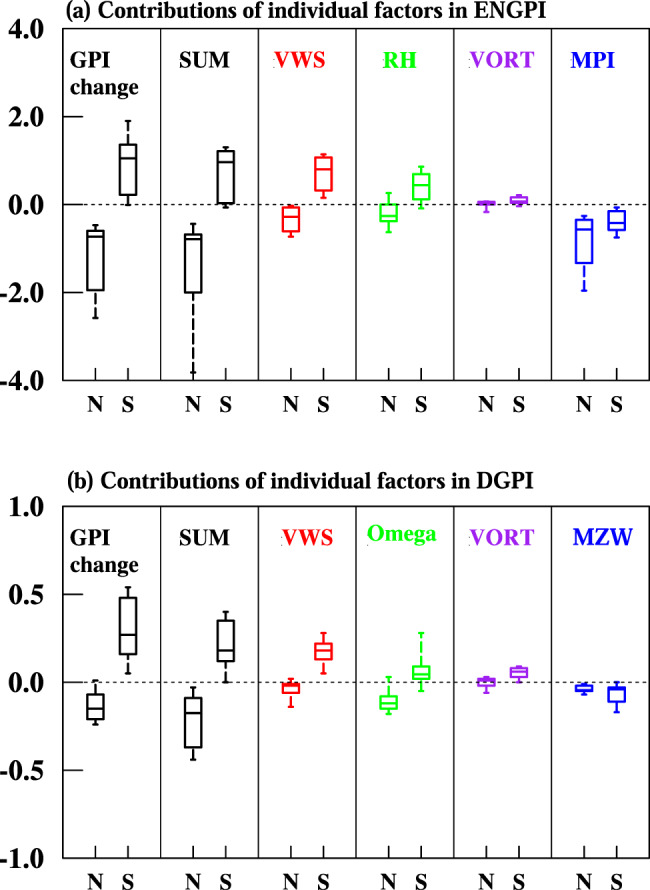
Contributions of individual large-scale environmental factors to the tropical cyclone (TC) genesis frequency change. The TC genesis frequency change is measured by the anthropogenic aerosol-induced hemisphere-averaged genesis potential indices (GPIs) between the modern period (1985–2014) and pre-Industrial period (1850–1879). **a** Emanuel-Nolan’s GPI (ENGPI), **b** dynamic GPI (DGPI). SUM means the sum of the four individual factors’ contribution. In ENGPI, the four factors are vertical wind shear (VWS), 600 hPa relative humidity (RH), 850 hPa absolute vorticity (VORT), and maximum potential intensity (MPI), respectively. In DGPI, the four factors include the VWS, mid-level pressure velocity (Omega), VORT, and meridional gradient of 500 hPa zonal wind (MZW). Whiskers, boxes, and the bars inside the boxes represent the 10^th^–90^th^ percentiles, quartile range, and median, respectively. The letters N and S represent the averages from July-October for the Northern Hemisphere and from January-April for the Southern Hemisphere, respectively.

Our analysis reveals an NH-SH asymmetric response of TC genesis frequency (TCGF) to the historical anthropogenic aerosol forcing (Fig. 1). The asymmetric response is characterized by an overall decrease of TCs during the NH TC peak season (July–October), whereas an increase and southward shift of TC formation during the SH TC peak season (January-April). The reduced NH TCGF occurs in all latitudes in North Pacific and North Atlantic. The TCGF response over the North Atlantic is consistent with the results from the ensemble simulations of a climate model with comprehensive aerosol effects and multiple coupled models with different complexity treatments of aerosol^14^. The increased SH TCGF is seen mainly in the major TC formation zone between 10°S and 25°S. The two TC proxies show consistent hemispherically asymmetric TCGF responses. In ENGPI, all models simulated reduced TCGF over the NH oceans [5°N–30°N, 110°E–20°W], and 11 out of 13 models (~85%) simulated an increased TCGF over SH oceans [10°S–25°S, 40°E-140°W] under anthropogenic aerosol forcing (Fig. 2). Similarly, in the DGPI, 11 out of 13 models simulated the reduction of TCGF over the NH oceans, and all models simulated the increased TCGF over the SH oceans (Fig. 2).

Changes in large-scale environmental conditions are arguably responsible for the asymmetric TCGF pattern. The vertical wind shear, mid-level moisture, and 500 hPa vertical pressure velocity consistently show the hemispherically asymmetric response under anthropogenic aerosol forcing (Supplementary Fig. 2a–f). Most of the models share an overall NH-enhanced and SH-weakened pattern of vertical wind shear, the NH-dry and SH-wet pattern of mid-troposphere, and NH-suppressed and SH-enhanced upward motion over 500 hPa over the TC formation zones. All these large-scale condition changes agree well with the hemispherically asymmetric TCGF distribution (Fig. 1). Note that the 600 hPa relative humidity and 500 hPa upward motion have similar patterns with a pattern correlation coefficient of 0.64, indicating that the mid-level upward motion in DGPI plays a similar role as the 600 hPa humidity in the ENGPI. Although favoring for reducing NH TC formation, the maximum potential intensity (MPI) and 500 hPa zonal shear vorticity failed to explain SH TC increase (Supplementary Fig. 2g–h). The 850 hPa absolute vorticity shows decreased cyclonic vorticity in both NH and SH TC formation zones, which positively contribute to the asymmetric TCGF pattern; however, its magnitude is small, suggesting a minor contribution (Supplementary Fig. 2i–j).

We quantify the relative importance of each large-scale environment condition using the GPI budget analyses (Methods, Supplementary Table 2). The relative contribution of an individual factor X is determined by comparing the GPI during the pre-Industrial period with the GPI calculated using the factor X’s modern (1985-2014) value while keeping the rest of the three factors at the pre-Industrial values. Figure 2 shows that over 90% (75%) models indicate that the vertical wind shear change increases (decreases) the SH (NH) TCs in both TC proxies. Over 75% of models suggest the mid-level moisture and 500 hPa vertical motion can cause an NH-decrease and SH-increase of TCGF pattern (Fig. 2). Other factors, the MPI and 500hPa zonal wind shear vorticity, contribute to decreased NH TCGF. The low-level absolute vorticity contributes to increased SH TCGF; however, their overall roles are minor (Fig. 2).

### Why anthropogenic aerosol causes the NH-decrease and SH-increase TCs

To understand the mechanism by which anthropogenic aerosol impacts global TC formation requests addressing the following fundamental question: How does the anthropogenic aerosol affect the global-scale TC formation environmental conditions, especially for the vertical wind shear and mid-level ascent/moisture? In theory, anthropogenic aerosol emission can affect the Earth’s energy balance through radiative forcing and microphysical effects^12,29,30^. Aerosols generally reduce the downwelling surface solar radiation by reflection, scattering, and absorption, therefore, cools the Earth’s surface.

**Fig. 3:**
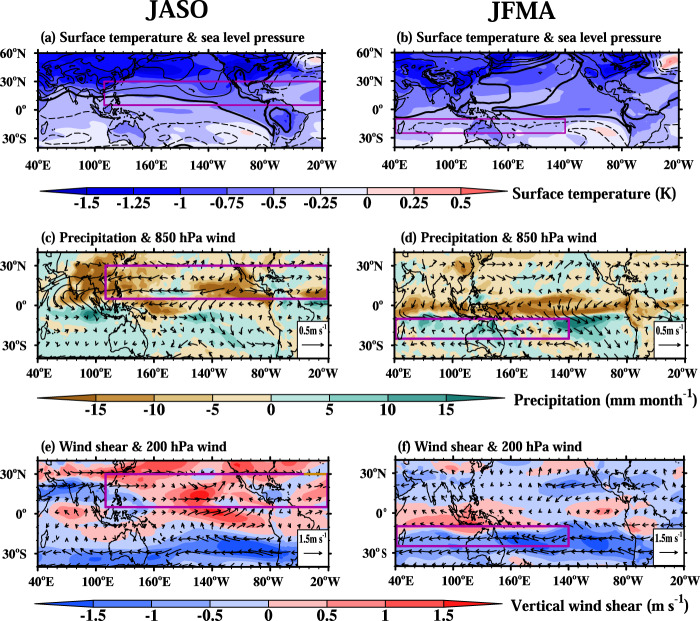
The impacts of anthropogenic aerosol on tropical cyclone (TC) environmental fields. The left and right panels show the changes in the Northern Hemisphere TC season (July-October, JASO) and the Southern Hemisphere TC season (January−April, JFMA) between the modern period (1985–2014) and pre-Industrial period (1850–1879), respectively. **a**, **b** surface temperature (shaded, K) and sea level pressure (contour, Pa). The solid and dashed lines indicate the positive and negative values, respectively, with a contour interval of 20 Pa. The zero lines are bolded. **c**, **d** 850 hPa circulation (m s^−1^) and precipitation (shaded; mm month^−1^). **e**, **f** 200 hPa circulation (m s^−1^) and vertical wind shear (magnitude of wind difference between 850 hPa and 200 hPa, shading, m s^−1^). The purple boxes denote the Northern Hemisphere and Southern Hemisphere tropical cyclone formation zones.

Figure 3 shows that the response of surface temperature to anthropogenic forcing is characterized by a more extensive cooling over the NH and less cooling over the SH in both boreal and austral summer, thus creating a significant interhemispheric temperature difference (ITD) (Fig. 3a, b). There is an evident weakening of climatological ITD in boreal summer and an opposite enhancement in austral summer. The ITD change can generate anomalous high pressures over the NH and low pressure over the SH (Fig. 3a, b), thus inducing low-level southward cross-equatorial flows (Fig. 3c, d) and the compensating upper-level northward cross-equatorial flows (Fig. 3e, f). These out-of-phase cross-equatorial flows indicate changes in the meridional overturning divergent circulation, which suppresses upward motion and precipitation in the NH TC formation zone during boreal summer, whereas increases upward motion and precipitation in the SH TC formation zone along the southern Indian Ocean and South Pacific convergence zones (Fig. 3c, d).

The aforementioned argument can be confirmed by energy balance. For instance, during boreal summer, the reduced ITD and weakened land-sea thermal contrast inhibit the NH monsoon’s development (Fig. 3c). From an energy perspective, the reduction of radiative forcing due to aerosol-radiation/aerosol-cloud interactions should be compensated by reduced precipitation latent heat release. This is demonstrated by suppressed precipitation over the NH and the weakened mid-level vertical velocity over the NH convergence zone (e.g., NH monsoon regions and oceanic convergence zone; Supplementary Fig. 2e). The anomalous ascending (descending) motion transport high (low) relative humidity air to the mid-troposphere, generating the hemispherically asymmetric mid-level moisture pattern. In summary, the consistent dynamic and energetic arguments explained how the anthropogenic aerosol forcing decreased (increased) the mid-tropospheric upward motion and associated relative humidity in the NH (SH) TC formation zone. Overall, the anthropogenic aerosol forced changes in vertical motion dynamically drive the hemispherical asymmetry of thermodynamic conditions, which benefits the NH-decreased and SH-increased TCGF pattern.

The cross-equatorial divergent circulation is affected by Earth’s rotation. The Coriolis force deflects the northward cross-equatorial flows eastward in the NH and westward in the SH. Thus, during the NH summer, the aerosol forcing generates easterly (westerly) at the low-level (upper-level), enhancing the vertical shear in the NH TC formation zone (Fig. 3c, e). This agrees well with the NH-increased meridional temperature gradient (Supplementary Fig. 3a, c). In contrast, during the SH summer, the aerosol forcing tends to decrease westerly (easterly) at the low-level (upper-level), reducing the vertical wind shear in the SH TC formation zone (Fig. 3d, f).

During austral summer, the anthropogenic aerosol forcing induces a weak cooling (~ −0.5^o^C) over the equatorial region. As such, precipitation is decreased over the equatorial region but increased over the off-equatorial region of the SH (Fig. 3d), implying a southward shift of the ITCZ, consistent with the previous theoretical work^31,32^. The decreased diabatic heating over the equatorial region weakens the upper-troposphere meridional temperature gradient, resulting in a weakening of 200 hPa westerly flow over the band 10°S–25°S (Fig. 3f and Supplementary Fig. 3b, d). It further decelerates the mean SH subtropical jet, resulting in the weakened vertical wind shear over the areas favoring SH TCs (Figs. 1 and 3f). This explains how the anthropogenic aerosol increased (decreased) vertical wind shear in the NH (SH) TC formation zones, thus suppressing (promoting) TC formation in the NH (SH).

**Fig. 4:**
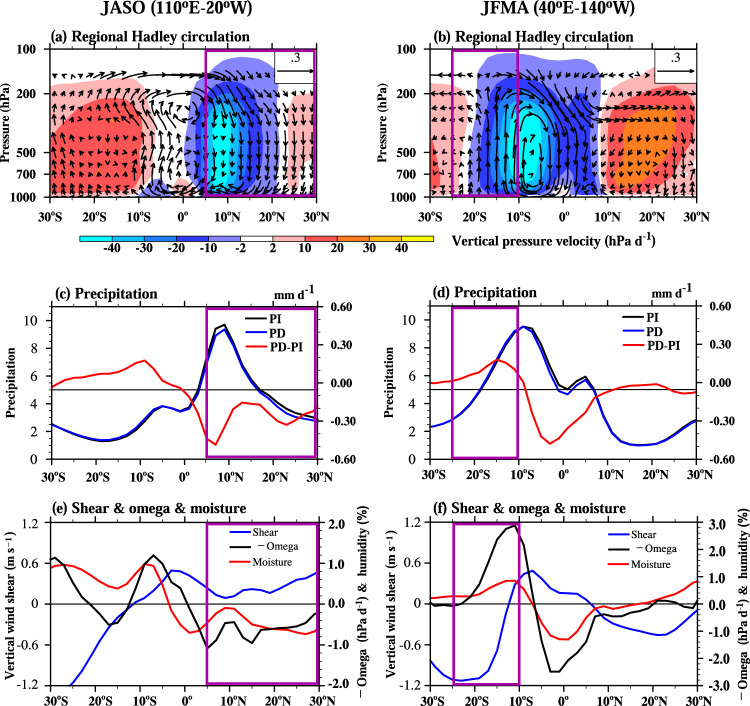
Anthropogenic aerosol-induced zonal mean responses in tropical cyclone (TC) seasons. The left and right panels show the zonal mean over 110°E–20°W during the Northern Hemisphere TC season (July-October, JASO) and 40°E–140°W during the Southern Hemisphere TC season (January–April, JFMA), respectively. **a**, **b** Climatological mean meridional circulation (regional Hadley circulation) and its changes. The shading indicates climatological vertical pressure velocity (hPa d^−1^) for pre-Industry period (1850–1879), and the vector is the composite of zonal wind change (m s^−1^) and 100 times of vertical velocity change (Pa s^−1^). **c**, **d** precipitation (mm month^−1^) for pre-Industrial period (PI, black), modern period (PD, blue), and their difference (PD-PI, red). **e**, **f** changes of vertical wind shear (blue, left y-axis, m s^−1^), negative vertical pressure velocity (black, right y-axis, hPa d^−1^), and 500 hPa relative humidity (red, right y-axis, %). The purple boxes mark the latitudinal extents of the TC formation zones.

The mechanisms explained can be reinforced by examining the changes of the zonal mean circulation averaged over the TC formation zones in the North Pacific and North Atlantic (110°E–20°W) for July–October and the SH oceans (40°E–140°W) for January–April (Fig. 4). The zonal mean meridional circulation is referred to as regional Hadley circulation.

During boreal summer, the climatological mean upward branch of the regional Hadley circulation and ITCZ are mainly located over 3°N-15°N, where the anthropogenic aerosol induces subsidence and suppressed precipitation (Fig. 4a, c). In contrast to the climatology of the regional Hadley circulation, the aerosol-induced low-level southward and upper-level northward across-equatorial flows weaken the lower and upper branches of the regional Hadley circulation due to the decreased diabatic heating over the NH monsoon zones (Figs. 3c and 4a). The downdraft associated with the weakened regional Hadley circulation transports the dry atmosphere air to the mid-troposphere, resulting in the low moisture over the lower-to-middle troposphere of NH tropics (Fig. 4e). The upper-level northward cross-equatorial flow deflects eastward due to the Coriolis force that accelerates the upper tropospheric zonal wind, contributing to the enhanced vertical wind shear over 5°N–30°N (Fig. 4e). Thus, the less favorable TC environment is readily seen over the NH oceans.

However, in the SH, the situation is different. During the austral summer, the aerosol-induced equatorial cooling produces strong descent and associated reduced precipitation near the equator (Fig. 4b, d). The southward flow from the equator to 15°S transports climatological high humidity air and enhances boundary layer moisture convergence, strengthening the ascending motion and precipitation around 15°S. Since the climatological ITCZ and updraft is located near 10°S, the anthropogenic aerosol-induced circulation anomaly tends to shift the regional Hadley circulation and ITCZ southward (Fig. 4b, d), increasing the upward motion in the SH TC formation zone. Meanwhile, the upward transport of high humidity air would increase the mid-level humidity in the TC formation zone. The substantial reduction of precipitation over the equatorial region contributes to the cooling over the equatorial troposphere, reducing the meridional temperature gradients, which decelerates the upper-tropospheric westerly (Supplementary Fig. 3c, d), resulting in decreased vertical wind shear. Therefore, over the SH TC formation zone, the aerosol forcing increases mid-tropospheric upward motion and relative humidity while decrease vertical wind shear (Fig. 4f), favoring TC formation in the SH.

## Discussion

We find that the historical anthropogenic aerosol has forced a hemispherically asymmetric TCGF pattern, namely, the NH-reduced and SH-increased TCGF (Fig. 1). We have determined that the anthropogenic aerosol affects TC formation in both hemispheres primarily through altering the large-scale vertical wind shear and the mid-tropospheric upward motion or relative humidity. The cause of the TC formation environmental condition changes is rooted in the anthropogenic aerosol-induced NH-cooler-than-SH pattern and the NH-increased and SH-decreased meridional temperature gradients (Fig. 3a, b). We first pointed out that the anthropogenic aerosol forcing increases the NH equator-to-polar temperature differences while reducing the SH counterpart. The increased NH temperature difference strengthens vertical wind shear, reducing NH TC genesis. The opposite is true for the SH. We also find that the anthropogenic aerosol-induced equatorial cooling during the austral summer (Fig. 3b) suppresses equatorial convection and enhances the upward motion in the SH TC formation zone, increasing SH TC formation.

**Fig. 5:**
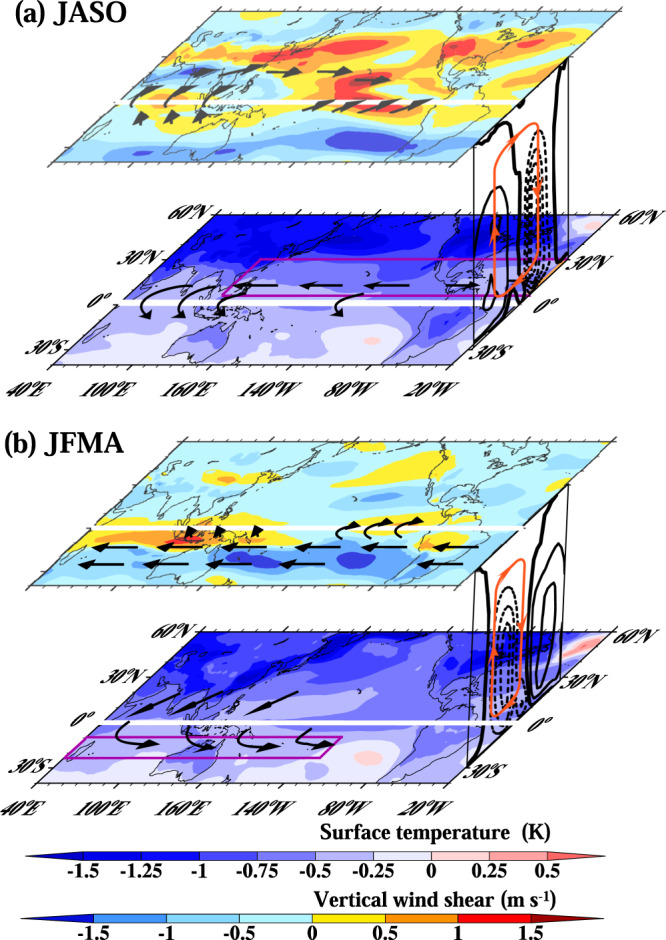
Schematic diagram illustrating the historical anthropogenic aerosol-forced circulation changes between the pre-Industrial period (1850–1879) and modern period (1985–2014). **a** Northern Hemisphere tropical cyclone (TC) season (July-October, JASO), **b** Southern Hemisphere TC peak season (January–April, JFMA). In each panel, the low-level map shows the changes of surface temperature (shaded, K). The upper-level map shows the change of vertical wind shear (shaded, m s^−1^). The arrows highlight the cross-equatorial flow and associated rotational flow changes. The right-side vertical cross-section shows the climatological Hadley cell measured by vertical pressure velocity (contours) and aerosol-induced change (red lines). The NH-cooler-than-SH pattern produces a low-level southward cross-equatorial transport of moist static energy, weakening the NH ascent while strengthening the SH ascent. The NH-increased and SH-decreased meridional temperature gradients strengthen vertical wind shear in the NH but reduce the vertical wind shear in the SH. The less (more) favorable TC formation environment suppresses (promotes) the formation of NH (SH) TCs.

Figure 5 highlights the aerosol-forced atmospheric circulation and precipitation changes that can alter the large-scale environmental conditions for TC formation. This schematic diagram is based on the results shown in Figs. 3 and 4. The processes are briefly summarized as follows. The anthropogenic aerosol cools the NH more than the SH in both boreal and austral summer, generating hemispheric surface pressure gradients and low-level cross-equatorial flows from NH to SH. These changes are particularly evident over the North Pacific and North Atlantic TC formation zone from 110°E–20°W and over the SH TC formation zone extending from 40°E–140°W (the boxed regions shown in Fig. 5). The low-level southward cross-equatorial flows transport water vapor and moist static energy from NH to SH, thereby weakening the NH boundary layer moisture convergence, thus the ITCZ and the Hadley cell updraft and intensity, whereas strengthening the SH ITCZ and associated Hadley cell. The weakened (strengthened) upward motion in the NH (SH) reduces (enhances) the upward transport of moist air, thus the mid-tropospheric humidity. Furthermore, the vertical wind shear increases in the NH TC formation zone due to the Earth’s rotation that deflects the upper-level northward cross-equatorial flows and accelerates upper-level westerly. In contrast, the vertical wind shear in the SH TC formation zone decreases due to the reduced SH meridional temperature gradients that decelerate upper-level westerlies.

Note that the anthropogenic aerosol-reduced precipitation and mid-level vertical motion are mainly located over the monsoon convergence zones, especially during boreal summer (Supplementary Fig. 2e). It means that the substantial weakening of vertical circulation over the monsoon region has a large contribution to the mean Hadley circulation change and ITCZ location shift. We, therefore, argue that both the changes in the global-scale TC formation and the Hadley circulation are responses to the anthropogenic aerosol forcing. This differs from prior studies that directly attribute TC formation change to ITCZ location movement^21–23^ or local Hadley circulation displacement^14^.

One may concern about whether the complexity of aerosol treatments exerts different impacts on TC activities^14,33^. As listed in Supplementary Table 1, four models used the prescribed aerosol forcing while nine models used the interactive aerosol module in the hist-aer experiment. The prescribed aerosol forcing considers only the effect of aerosol optical properties and their associated Twomey effect^34^. The interactive aerosol module could fully describe their impact on radiation and cloud microphysical process and cloud amount. Both model groups show a consistent NH-decrease and SH-increase of TCGF, but the interactive aerosol group offers more SH TCs (Supplementary Fig. 4). The more significant SH TCGF (Supplementary Fig. 4) is caused by the same physical mechanism mentioned above and may be attributable to the more prominent surface temperature response (Supplementary Fig. 5a, b). The interactive aerosol models produce a sharper NH-SH thermal contrast, which more significantly increases upward motion and reduces the vertical wind shear in the SH TC genesis zone (Supplementary Fig. 5c–f), leading to the more SH TCs. Open questions remain on whether the microphysical effect of aerosol may affect the TC development. Studies suggested microphysical effect of aerosol could weaken TC intensity by causing the decrease of inner-core TC rainband^33,35^. Thus, the microphysical effect of aerosol may be unfavorable for TC genesis. Nevertheless, TCs often generate over the oceans where far away from the continent. The extensive tropical precipitation may significantly wash out the aerosols before reaching the TC development zone, limiting the microphysical effect of aerosols. High-resolution models with an explicit treatment of aerosol microphysical effect would be an advanced tool to explore the detailed mechanism and its associated effect.

The finding and understanding gained from this study may help to constrain the uncertainty in the TCGF projection. Higher-resolution coupled model and TC-permission models’ simulations can directly detect the simulated TC-like vortex. The use of such models is a more desirable strategy for further quantification of the anthropogenic aerosol-induced TC change over a global scale and regional scale. This study highlights the possible connection between the NH anthropogenic aerosol emission and SH TC activity. The particular high emission of anthropogenic aerosol as treated in Shared Socioeconomic Pathways 3-7.0^36^ may further increase the SH TC threat. On the other hand, with reduced anthropogenic aerosol emissions, the continuing greenhouse gases emission could more significantly increase the NH TC threat.

## Methods

### CMIP6 model and simulations

 The hist-aer experiments from 13 CMIP6 models are used to explore the impacts of anthropogenic aerosol forcing on TCGF (Supplementary Table 1). In the Detection and Attribution MIP of CMIP6, the hist-aer experiment is designed to identify the impacts of anthropogenic aerosol. Its experimental design resembles the CMIP6 historical simulation but is forced by the temporal evolution of historical anthropogenic aerosol forcing only^26^. The detailed information of the 13 models and the treatment aerosol are listed in Supplementary Table 1. To identify the influence of historical anthropogenic aerosol emission on changes in TC climatology, two sub-periods of 1850–1879 and 1985–2014, respectively, are selected to represent the pre-Industrial period and modern period. The use of the 30-year climatology helps suppress the uncertainties that arise from the internal variability within the coupled climate system to identify the impacts of anthropogenic aerosol better. All outputs are re-gridded to the uniform resolution of 2° × 2° in latitudinal and longitudinal directions by bilinear interpolation to facilitate analyses.

### Diagnostic analysis of genesis potential index

Two GPIs have been used to investigate dynamic and thermodynamic factors affecting TCs. The ENGPI consists of four environmental factors that control TC formation: the vertical wind shear between 850 and 200 hPa, the 850 hPa absolute vorticity, the 600 hPa relative humidity, and the maximum potential intensity (MPI)^27^. The first two are dynamic factors, and the latter two are thermodynamic factors. The ENGPI^27^ is expressed as:

1 $${{{{{\rm{ENGPI}}}}}}={|{10}^{5}\eta |}^{\frac{3}{2}}{\left(\frac{{{{{{\rm{H}}}}}}}{50}\right)}^{3}{\left(\frac{{{{{{\rm{MPI}}}}}}}{70}\right)}^{3}{\left(1+0.1{{{{{{\rm{V}}}}}}}_{{{{{{\rm{shear}}}}}}}\right)}^{-2}$$

where ${{{{{\rm{\eta }}}}}}$ is the 850 hPa absolute vorticity, H is the 600 hPa relative humidity, ${{{{{{\rm{V}}}}}}}_{{{{{{\rm{shear}}}}}}}$ is the 200–850 hPa wind shear value. The MPI is the theoretical upper bound on TC intensity under a given set of atmospheric and oceanic conditions and is calculated with the following expression^37^:

2 $${{{{{{\rm{MPI}}}}}}}^{2}=\frac{{{{{{{\rm{T}}}}}}}_{{{{{{\rm{s}}}}}}}}{{{{{{{\rm{T}}}}}}}_{0}}\frac{{{{{{{\rm{C}}}}}}}_{{{{{{\rm{k}}}}}}}}{{{{{{{\rm{C}}}}}}}_{{{{{{\rm{D}}}}}}}}[{{{{{{\rm{CAPE}}}}}}}_{{{{{{\rm{MS}}}}}}}-{{{{{{\rm{CAPE}}}}}}}_{{{{{{\rm{M}}}}}}}]$$

where ${{{{{{\rm{T}}}}}}}_{{{{{{\rm{S}}}}}}}$ is SST (^o^C), ${{{{{{\rm{T}}}}}}}_{{{{{{\rm{o}}}}}}}$ is the mean outflow temperature (^o^C), ${{{{{{\rm{C}}}}}}}_{{{{{{\rm{K}}}}}}}$ is the exchange coefficient for enthalpy, and ${{{{{{\rm{C}}}}}}}_{{{{{{\rm{D}}}}}}}$ is the drag coefficient. ${{{{{{\rm{CAPE}}}}}}}_{{{{{{\rm{MS}}}}}}}$ is the convective available potential energy (CAPE) for an air parcel brought to saturation at the radius of maximum winds. ${{{{{{\rm{CAPE}}}}}}}_{{{{{{\rm{M}}}}}}}$ is the CAPE of a parcel brought to the radius of maximum winds without the input of energy or moisture.

The DGPI consists of four dynamic factors: 500 hPa vertical pressure velocity, the vertical wind shear, the 850 hPa absolute vorticity, and 500 hPa zonal wind-induced shear vorticity^28^. The DGPI was derived from both the present and future global warming environments, providing a complementary measure to ENGPI. The formulation of DGPI^28^ is:

3 $${{{{{\rm{DGPI}}}}}}={(2+0.1{{{{{{\rm{V}}}}}}}_{{{{{{\rm{shear}}}}}}})}^{-1.7}{\left(5.5-\frac{\partial {{{{{\rm{u}}}}}}}{\partial {{{{{\rm{x}}}}}}}{10}^{5}\right)}^{2.3}{(5-20\omega )}^{3.3}{(5.5+|{10}^{5}\eta |)}^{2.4}{{{{{{\rm{e}}}}}}}^{-11.8}-1$$

where $\frac{\partial {{{{{\rm{u}}}}}}}{\partial {{{{{\rm{x}}}}}}}$ is the meridional gradient of zonal wind at 500 hPa, and ${{{{{\rm{\omega }}}}}}$ is the 500  hPa vertical pressure velocity. Both indices can well represent the climatological mean of observed and CMIP6 model simulated TCGF distribution (Supplementary Fig. 1). Best-track data were taken from the International Best Track Archive for Climate Stewardship (IBTrACS) v03r05^38^. In this study, GPI budget analysis is used to understand the key factors (e.g., MPI, vertical wind shear, mid-troposphere relative humidity, and low-level vorticity) associated with anthropogenic aerosol forcing in modulating global TCs. To examine the relative importance of the four factors to the aerosol-induced changes in GPI, we performed a single factor perturbation analysis^39,40^. For example, to identify MPI’s role, we recalculated the GPI using modern climatological mean MPI and hold the other three variables as the PI values. The difference between the recalculated GPI and GPI in PI is estimated as the possible impact of MPI in response to anthropogenic aerosol forcing changes. The detailed design of all budget analyses is shown in Supplementary Table 2. We mainly focus on the response of TCs over both hemispheres; thus, the hemispheric mean of quantities is calculated over the TC main development region ([5°N–30°N, 110°E–20°W] of the NH and [10°S–25°S, 40°E–140°W] of the SH) during the TC peak season of each hemisphere (July-October for the NH and January-April for the SH).

## Supplementary information

Supplementary Information

## Data Availability

All data used in this study are available in public repositories. The CMIP6 simulations are available at https://esgf-node.llnl.gov/search/cmip6/. The IBTrACS data is available at https://www.ncdc.noaa.gov/ibtracs/.

## Code availability

The codes that support the findings of this study are routinely and commonly used in the community, which are fully available from the first author (jianc@nuist.edu.cn) upon reasonable request.
