# Author Correction: Hemisphere-asymmetric tropical cyclones response to anthropogenic aerosol forcing

**DOI:** 10.1038/s41467-021-27888-z

**Published:** 2022-01-07

**Authors:** Jian Cao, Haikun Zhao, Bin Wang, Liguang Wu

**Affiliations:** 1 Key Laboratory of Meteorological Disaster, Ministry of Education/Joint International Research Laboratory of Climate and Environment Change/Collaborative Innovation Center on Forecast and Evaluation of Meteorological Disasters, Nanjing University of Information Science and Technology, Nanjing, China; 2 Earth System Modeling Center, Nanjing University of Information Science and Technology, Nanjing, China; 3 Department of Atmospheric Sciences, University of Hawaii at Mānoa, Honolulu, HI USA; 4 Department of Atmospheric and Oceanic Sciences and Institute of Atmospheric Sciences, Fudan University, Shanghai, China

**Keywords:** Atmospheric dynamics, Climate change

Correction to: *Nature Communications* 10.1038/s41467-021-27030-z, published online 22 November 2021.

The original version of this Article contained an error in ref. 5, which was incorrectly given with the wrong first author name as: Carmago, S. J. The correct form of ref. 5 is: Camargo, S. J. Global and regional aspects of tropical cyclone activity in the CMIP5 models. *J. Clim*. **26**, 9880–9902 (2013).

This has been corrected in the PDF and HTML versions of the Article.

