# Supplementary material for: Hemisphere-asymmetric tropical cyclones response to anthropogenic aerosol forcing
Source: Nat Commun. 2021 Nov 22;12:6787. doi: 10.1038/s41467-021-27030-z (PMC8608990; doi:10.1038/s41467-021-27030-z)
Supplement: Supplementary file 1 — Supplementary Information [file 41467_2021_27030_MOESM1_ESM.pdf]

Supplementary information for

Hemisphere-asymmetric Tropical Cyclones Response to Anthropogenic Aerosol Forcing

Cao, Jian[1,2], Haikun Zhao[1], Bin Wang[2,3], Liguang Wu[4]

[1]*Key Laboratory of Meteorological Disaster, Ministry of Education/Joint International Research Laboratory of Climate and Environment Change/Collaborative Innovation Center on Forecast and Evaluation of Meteorological Disasters, Nanjing University of Information Science and Technology, Nanjing, China*

[2]*Earth System Modeling Center, Nanjing University of Information Science and Technology, Nanjing, China*

[3]*Department of Atmospheric Sciences, University of Hawaii at Mānoa, Honolulu, HI, USA*

[4]*Department of Atmospheric and Oceanic Sciences and Institute of Atmospheric Sciences, Fudan University, Shanghai, China*

Contents of this file

Supplementary Tables 1-2

Supplementary Figures 1-5

Introduction

Tables and Figures below provide supporting materials to substantiate the results and discussions presented in the main text.

Supplementary Table 1. Description of 13 CMIP6 models used in this study

Institution	Model acronym	Atmosphere resolution Lon x Lat	Aerosol
Australian Community Climate and Earth System Simulator (ACCESS)	ACCESS-ESM1-5	192 x 144	interactive
Beijing Climate Center, China Meteorological Administration	BCC-CSM2-MR	320 x 160	prescribed
National Center for Atmospheric Research (NCAR)	CESM2	288 x 192	interactive
Canadian Centre for Climate Modelling and Analysis (CCCma)	CanESM5	128 x 64	interactive
Centre National de Recherches Météorologiques (CNRM)	CNRM-CM6-1	256 x 128	interactive
Chinese Academy of Sciences (CAS)	FGOALS-g3	180 x 80	prescribed
Geophysical Fluid Dynamics Laboratory (NOAA GFDL)	GFDL-ESM4	288 x 180	interactive
Goddard Institute for Space Studies	GISS-E2-1-G	90 x144	prescribed
Met Office Hadley Center	HadGEM3-GC31-LL	192 x 144	interactive
Institute Pierre-Simon Laplace (IPSL)	IPSL-CM6A-LR	144 x 143	prescribed
Agency for Marine-Earth Science and Technology (MIROC)	MIROC6	256 x 128	interactive
Meteorology Research Institute (MRI)	MRI-ESM2-0	320 x 160	interactive
Bjerknes Center for Climate Research (BCCR)	NorESM2-LM	144 x 96	interactive

Supplementary Table 2. Design of genesis potential index (GPI) sensitivity experiment. Shown are the results from Emanuel-Nolan's GPI and dynamic GPI (in the parenthesis). In the CTL-PI experiment, the GPI is calculated with the four factors from PI climatology (1850-1879). The GPI calculation in the CTL-Aerosol experiment used the climatological mean value from 1985-2014 of hist-aer experiment. The Sen-VWS(VWS), Sen-RH(Omega), Sen-VORT(VORT), and Sen-MPI(ZS) experiments are designed as CTL-PI experiment, except that the vertical wind shear, relative humidity (500 hPa omega), 850hPa vorticity, and MPI (meridional gradient of 500 hPa zonal wind, MZW) are replaced by the averaged value from 1985-2014 of hist-aer experiment, respectively. The difference between Sen-MPI and Sen-CTL is regarded as the contribution of MPI, so as other factors. The acronym of VWS, RH, and VORT represent vertical wind shear, mid-troposphere humidity, low-level vorticity. PI and Modern represent the climatologies of 1850-1879 and 1985-2014 from hist-aer experiment, respectively.

Exp. name	VWS(VWS)	RH(Omega)	VORT(VORT)	MPI(MZW)
CTL-PI	PI	PI	PI	PI
CTL-Aerosol	Modern	Modern	Modern	Modern
Sen-VWS(VWS)	Modern	PI	PI	PI
Sen-RH(Omega)	PI	Modern	PI	PI
Sen-VORT(VORT)	PI	PI	Modern	PI
Sen-MPI(MZW)	PI	PI	PI	Modern

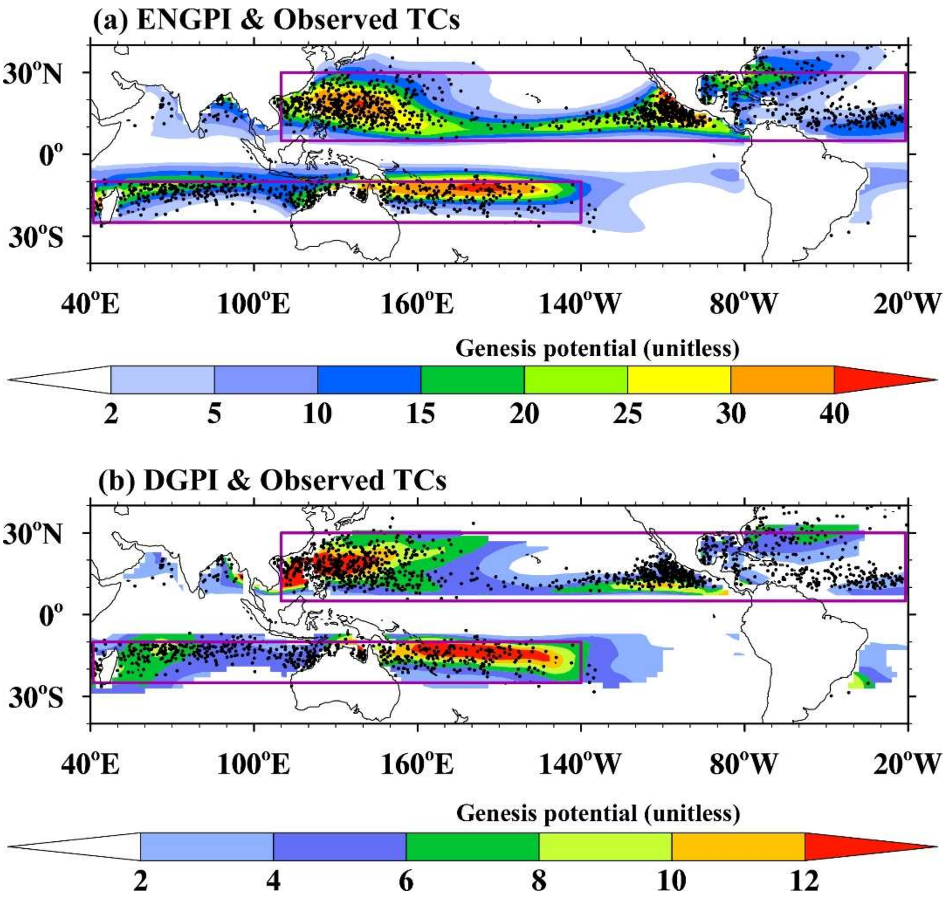

Supplementary Figure 1. Comparison of observed tropical cyclone (TC) genesis location with those estimated by genesis potential indices (GPIs) derived from CMIP6 historical experiment during 1985-2014. Shading indicates (a) the Emanuel-Nolan's GPI (ENGPI) and (b) dynamic GPI (DGPI), respectively. Best-track data were taken from the International Best Track Archive for Climate Stewardship (IBTrACS) v03r05. The purple boxes present the TC formation zones over the Northern Hemisphere and Southern Hemisphere. Dotted points are the observed TC genesis locations.

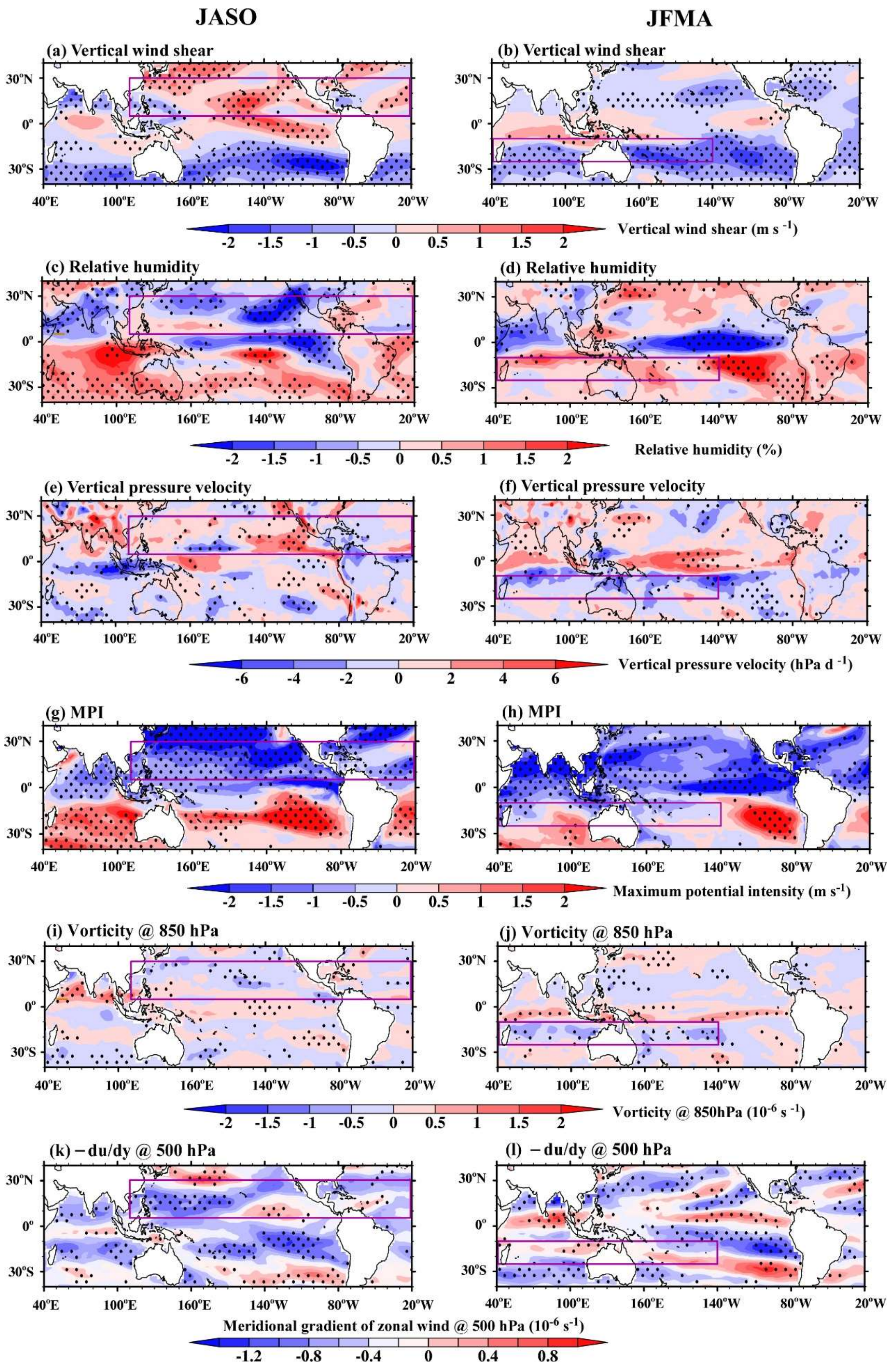

Supplementary Figure 2. The impacts of anthropogenic aerosol on tropical cyclone (TC) environmental fields. The left and right panels show the changes in the Northern

Hemisphere TC season (July-October, JASO) and the Southern Hemisphere TC season (January-April, JFMA) between the present-day (1985-2014) and pre-Industrial period (1850-1879) simulated by the hist-aer experiment from the 13 CMIP6 models, respectively. (a, b) vertical wind shear (m s^{-1}), (c, d) 600 hPa relative humidity (%), (e, f) 500 hPa vertical pressure velocity (hPa d^{-1}), (g, h) maximum potential intensity (MPI, m s^{-1}), (i, j) 850 hPa vorticity (10^{-6} s^{-1}), and (k, l) 500 hPa zonal wind shear vorticity (10^{-6} s^{-1}). Stippled regions indicate the sign changes are the same in at least 66% of models. The purple boxes present the TC formation zones over the Northern Hemisphere and Southern Hemisphere.

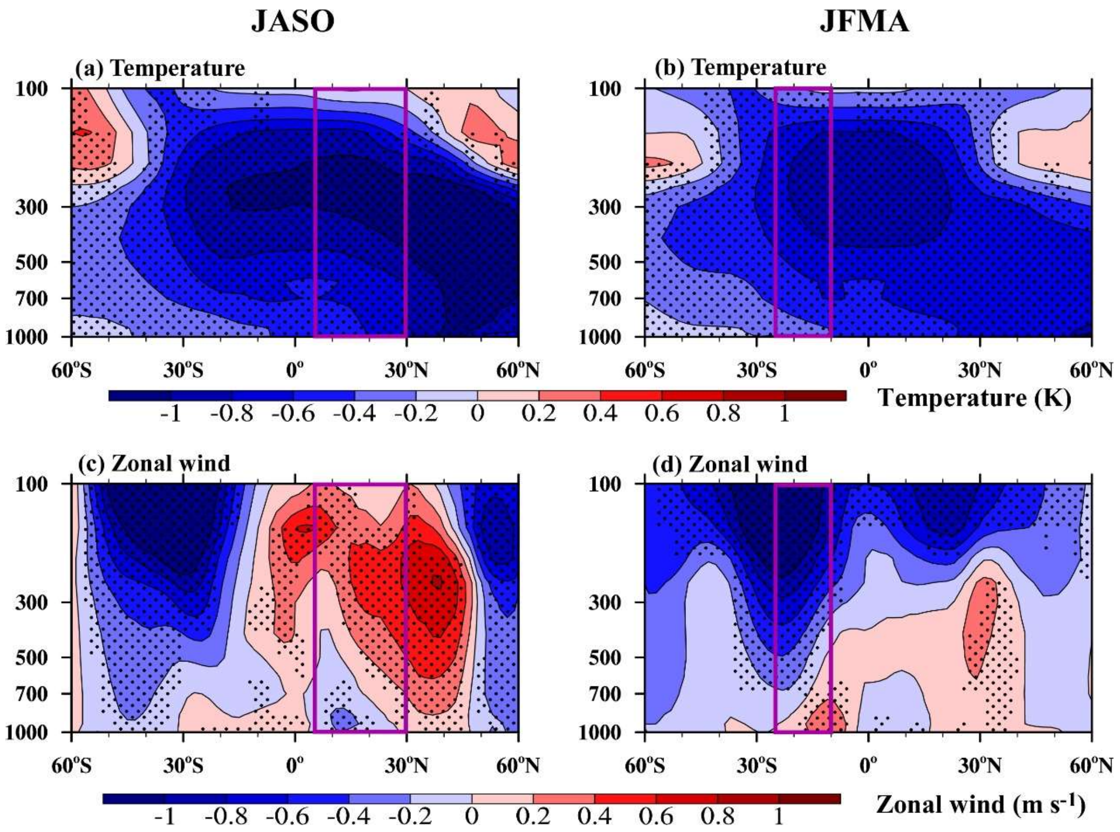

Supplementary Figure 3. Anthropogenic aerosol-induced changes of zonal mean air temperature and zonal wind speed between the modern period (1985-2014) and pre-Industrial period (1850-1879). (a) air temperature (K) for July-October (JASO) averaged over 110°E-20°W. (b) air temperature (K) for January-April (JFMA) averaged over 40°E-140°W. (c) the same as (a), but for zonal wind speed (m s^{-1}). (d) the same as (b), but for zonal wind speed (m s^{-1}). Stippled regions indicate the sign changes are the same in at least 11 of 13 (~85%) models. The purple boxes mark the latitudinal extents of the tropical cyclone formation zones.

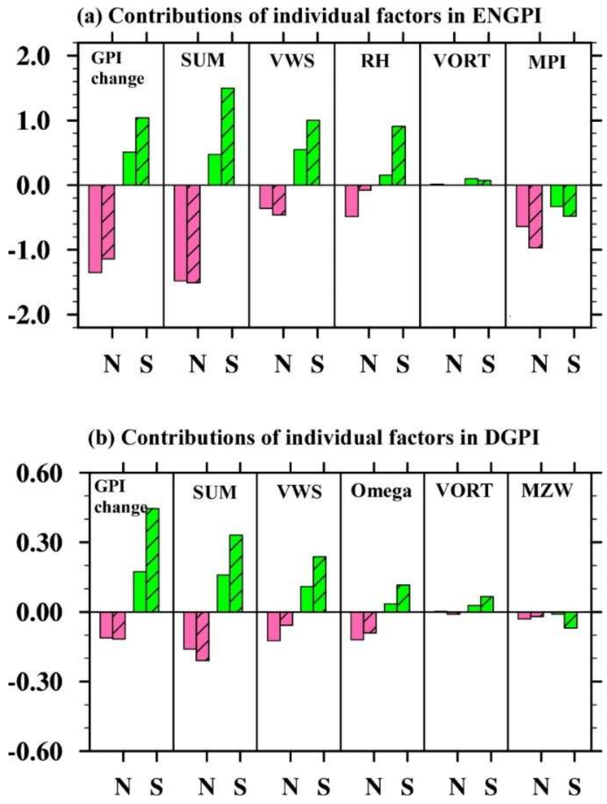

Supplementary Figure 4. Contributions of individual large-scale environmental factors to the tropical cyclone (TC) genesis frequency change in prescribed and interactive aerosol models. The TC genesis frequency change is measured by the anthropogenic aerosol-induced hemisphere-averaged genesis potential indices (GPIs) for prescribed (shaded) and interactive aerosol models (hatched) between the modern period (1985-2014) and pre-Industrial period (1850-1879). (a) Emanuel-Nolan's GPI (ENGPI), (b) dynamic GPI (DGPI). SUM means the sum of the four individual factors' contribution. In ENGPI, the four factors are vertical wind shear (VWS), relative humidity (RH), and low-level vorticity (VORT), and maximum potential intensity (MPI). In DGPI, the four factors include VWS, mid-level pressure velocity (Omega), VORT, and meridional gradient of 500 hPa zonal wind (MZW). The letters N and S represent the averages for the Northern Hemisphere TC season (July-October) and for the Southern Hemisphere TC season (January-April), respectively.

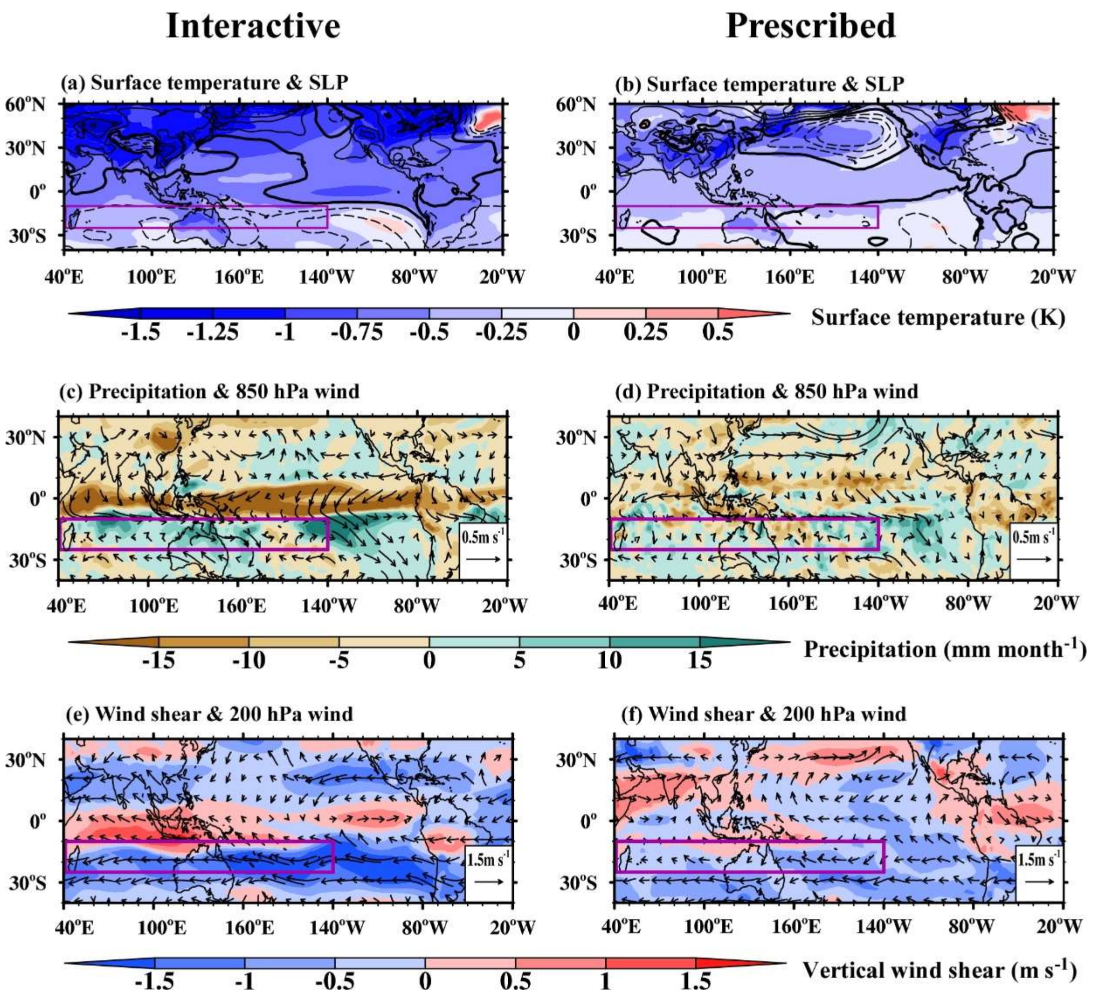

Supplementary Figure 5. Anthropogenic aerosol-induced tropical cyclone (TC) environmental fields changes during January-April. (a, b) surface temperature (shaded, K) and sea level pressure (SLP; solid and shaded lines indicate the positive and negative values, respectively, with a contour interval of 20 Pa. The zero lines are bolded), (c, d) 850 hPa circulation (m s^{-1}) and precipitation (shaded, mm $month^{-1}$), and (e, f) 200 hPa circulation (m s^{-1}) and vertical wind shear (shaded, m s^{-1}) for (left panel) interactive and (right panel) prescribed aerosol forcing models, respectively. The purple boxes present the TC formation zones over the Northern Hemisphere and Southern Hemisphere.
